# Can Cell-Free DNA in the Culture Medium Predict the Chromosomal Constitution of Preimplantation Embryos? Final Results from a Multicenter Study with 2539 Blastocysts

**DOI:** 10.3390/genes17040416

**Published:** 2026-03-31

**Authors:** Luis Navarro-Sánchez, Denny Sakkas, Nilo Frantz, Emilio de la Fuente Lucena, William Venier, Daria Maria Soscia, Gerardo Barroso, Claudio Bisioli, Michael DiMattina, Bilgen Teke, Luis Ernesto Escudero, Carmen Rubio

**Affiliations:** 1 Research and Development Department, Igenomix, Part of Vitrolife Group, 46980 Paterna, Spain; 2 IVIRMA Global Research Alliance, Waltham, MA 02451, USA; 3 Nilo Frantz Reproductive Medicine, Porto Alegre 91330-000, Brazil; 4 San Diego Fertility Center, San Diego, CA 92108, USA; 5 IVIRMA Global Research Alliance, Genera, 00197 Roma, Italy; 6 Department of Biomedicine and Prevention, University of Tor Vergata, 00133 Roma, Italy; 7 Nascere, Mexico City 05120, Mexico; 8 Pregna Medicina Reproductiva, Buenos Aires 1425, Argentina; 9 Dominion Fertility, Arlington, VA 22203, USA; 10 Bahçeci Health Group, 34394 Istanbul, Turkey; 11 Inmater Fertilidad, Lima 15036, Peru

**Keywords:** cfDNA, niPGT-A, SBM, concordance, clinical outcome, prioritization, inner cell mass, aneuploidy, blastocyst, non-invasive

## Abstract

Background/Objectives: In the last decade, non-invasive methods for aneuploidy detection have been explored. The most successful approach involves analyzing the cell-free DNA (cfDNA) released by the embryo into the culture medium. The main objective of this study is to examine the technical feasibility of this new approach called non-invasive PGT-A or niPGT-A. In addition, as an exploratory objective, the impact of the niPGT-A results on clinic outcomes will be assessed. Methods: This was a multicenter, international study that included 716 patients and 2539 blastocysts (ClinicalTrials.gov: NCT03520933). Each embryo was cultured following a specific protocol for niPGT-A. Individual spent blastocyst medium (SBM) and trophectoderm (TE) biopsy were obtained, analyzed, and compared to assess concordance. In a subset of embryos, the comparison also included an inner cell mass (ICM) biopsy. Clinical outcomes from the embryo transfers performed (all based on the TE result) were registered, and results were analyzed blindly regarding the impact of aneuploidies in the culture medium. Results: The concordance rate between SBM and TE was 79.1% (range: 74.1–82.1; cycles with autologous oocytes). This value increased to 87.0% when comparing SBM and ICM. Applying an adapted embryo culture protocol to collect the SBM for niPGT-A did not affect blastocyst quality. Analysis of the embryo transfers performed (*n* = 265) revealed a trend towards lower miscarriage rate in blastocysts where both TE and SBM were concordant and euploid (13.0%), compared to blastocysts with a euploid TE and an aneuploid SBM (22.2%). Conclusions: The results obtained show a high concordance between the SBM and TE biopsies. Although additional refinement of the technique would further increase niPGT-A’s performance, the results obtained support the potential use of this non-invasive approach for aneuploidy detection. The high concordance of the cfDNA present in the SBM with the corresponding ICM biopsy and the miscarriage rate observed in cases with an aneuploid SBM, despite the euploid TE results, also support niPGT-A’s capacity to assess embryo aneuploidies and its potential as a prioritization system for selecting blastocysts to transfer. This approach could hold special interest in patients with no PGT-A indications, couples that prefer not to biopsy their embryos or those who do not have access to invasive PGT-A.

## 1. Introduction

Over the past 50 years, assisted reproduction has evolved significantly, enabling an increasing number of individuals to fulfill their dream of having a healthy newborn at home. This remarkable progress has been achieved by the development of technical and scientific solutions to overcome the numerous challenges that arise throughout the patient’s journey. The effort has been multidisciplinary, covering relevant areas spanning the fields of gynecology, genetics, or molecular biology but also extending to cutting-edge perspectives such as automation, artificial intelligence, and non-invasiveness. Despite these advances, embryo selection—identifying the embryo with the highest likelihood of resulting in a healthy baby—remains a significant challenge.

Traditionally, embryo morphology has represented the most widely applied selection method. However, this approach cannot guarantee the selection of a euploid embryo, free of chromosomal aneuploidies (gains and losses of whole chromosomes or regions of them) [1]. As a consequence, invasive diagnostic techniques, particularly trophectoderm (TE) biopsy and preimplantation genetic testing for aneuploidies (PGT-A), have become standard approaches, enabling the high-resolution analysis of all chromosomes. By enabling the identification of euploid embryos, PGT-A enhances implantation potential, shortens the time to pregnancy, and reduces the incidence of miscarriage, which holds particular relevance to patients of advanced maternal age [2,3].

More recently, research groups have been working to develop non-invasive alternatives for blastocyst selection, such as those based on the analysis of morphokinetics (specific parameters or the global embryo development [4]), but also on the analysis of the spent embryo culture media, which contains DNA, RNA, and metabolites released by the blastocyst [5]. Among these molecules, DNA remains the most extensively evaluated and has achieved the greatest success to date. The technique that analyzes the embryonic cell-free DNA (cfDNA) is called non-invasive PGT-A or niPGT-A and has seen significant advances over its decade of development, progressing from bench research to clinical application and commercial availability.

The presence of embryonic cfDNA in spent blastocyst media (SBM) was first reported by Stigliani et al. in 2013–2014 [6,7]. Shortly afterwards, in 2016, two additional research groups published their results after analyzing SBM—that would otherwise be discarded as part of the standard embryo culture routine—to determine the embryo’s chromosomal status, specifically for aneuploidy detection [8,9]. Subsequent studies focused on the technical development of niPGT-A, aiming to establish optimal embryo culture protocols and methods for SBM analysis and results interpretation [10,11]. Factors such as the time of media collection [12,13,14,15], the need to perform additional embryo handling to increase the amount of cfDNA released [16,17] or contamination control [18] have been studied to obtain the highest SBM informativity rate and the highest concordance with other samples from the same embryo (either TE or inner cell mass (ICM) biopsies or even whole blastocysts (WB) [19,20]).

In this report, we present the final results of an extensive multicenter study on niPGT-A which started in 2018. An interim analysis, encompassing 1301 blastocysts, was published in 2020 [21]. This previous study provided promising results, supporting the technical validity of this new technique. We obtained high SBM informativity and concordance with TE biopsies. In addition, further analysis of the interim data was conducted, focused on assessing the effect that the minor but necessary modifications required to perform niPGT-A may have on embryo development. Most importantly, no negative effects on blastocyst quality and potential were observed, confirming the robustness of the embryo culture protocol used [22].

The objectives of this manuscript are to further assess the technical feasibility of niPGT-A with an expanded sample size after study completion, provide a more comprehensive analysis of SBM informativity and concordance rates, and assess the potential influencing factors to further validate our previous findings. Additionally, we aim to investigate cfDNA origin and explore the potential impact of niPGT-A results on clinical outcomes in patients undergoing IVF.

## 2. Materials and Methods

### 2.1. Study Design

A prospective, observational, multicenter study was conducted to assess the feasibility of analyzing the embryonic cell-free DNA released into the culture medium as a non-invasive method for aneuploidy detection. Details of an interim analysis of the study have been previously published [21]. In brief, patients undergoing PGT-A were recruited and their embryos were cultured following a modified protocol. On day 6, individual culture media drops were aspirated, and blastocysts were biopsied and vitrified as part of the PGT-A cycle. Euploid blastocysts (according to the TE biopsy results) were transferred in a subsequent frozen embryo deferred transfer cycle. The SBM samples were analyzed blindly and the results were compared to the TE biopsy findings and correlated with clinical outcomes. In a subset of patients who donated their embryos for research, an additional ICM biopsy was collected, and the results were compared for concordance with TE and SBM.

### 2.2. Ethical Approval

Ten IVF centers participated in the study. The study protocol was approved by an ethics committee at each of them. Detailed information on the ethical approvals is provided in Supplemental Table S1. The study protocol was registered at ClinicalTrials.gov (NCT03520933).

### 2.3. Study Participants

From April 2018 to December 2022, women aged 20–44 years undergoing PGT-A for various indications were recruited at the 10 participating centers in Europe, North and South America, and Asia. Ovum and sperm donation cases were included. Fertilization techniques were intracytoplasmic sperm injection (ICSI) or in vitro fertilization (IVF) in fresh oocytes; the sperm used was fresh or frozen. Carriers of structural abnormalities or monogenic diseases were excluded. Patients who fulfilled the inclusion criteria provided their written informed consent to participate in the study.

**Figure 1:**
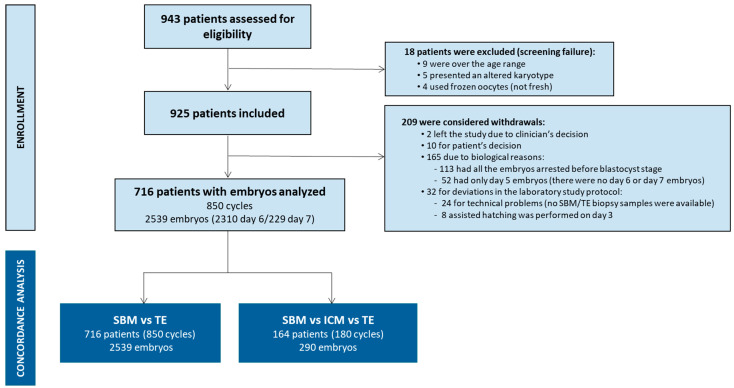
Study workflow. Patients included and samples analyzed.

Of the 943 patients assessed for eligibility, 716 were included, corresponding to 850 PGT-A cycles, from which 2539 day 6/7 blastocysts were obtained. Only patients and embryos in full compliance with the protocol were included, and all off-protocol cases were excluded prior to analysis. In addition, ICM’s contribution to the embryonic cfDNA in spent media was assessed in 290 blastocysts donated for research at 2 of the 10 participating centers (Boston IVF and Nilo Frantz e Centro de Reproduçao Humana). Figure 1 provides a detailed description of the study workflow, encompassing included or excluded patients (and the corresponding reasons), and the type and number of samples analyzed.

### 2.4. IVF Cycle

Controlled ovarian stimulation (COS) and induction of ovulation were performed using standard protocols at each site based on female age, basal hormone levels, basal ovarian reserve, and body mass index. The COS protocol was determined by the enrolling physician at each participating IVF clinic. ICSI or IVF was performed according to the protocols of the participating sites. Embryos were cultured in the regular culture media and bench-top incubators used in each IVF laboratory but according to the study culture protocol required to allow SBM collection. The different culture media used were single step—Irvine Scientific (Santa Ana, CA, USA), Life Global (Trumbull, CT, USA) or Vitrolife (Gothenburg, Sweden)—or sequential—Sage (Trumbull, CT, USA). Embryo culture was carried out in incubators with a controlled atmosphere containing 6–7% CO_2_ and 5–6% O_2_ (MINC, Cook Medical, Bloomington, IN, USA; MIRI, ESCO, Egaa, Denmark; C-top, Labotec, Göttingen, Germany; ASTEC, Astec, Minamizato, Japan; K-System G185/G210, CooperSurgical, Målov, Denmark) (Supplemental Table S2).

### 2.5. Embryo Culture, SBM Collection, TE Biopsy, and Blastocyst Vitrification

Embryos were cultured following the standard protocol of each IVF laboratory until day 4. On day 4, each embryo was washed using a new capillary in an individual series of 6 fresh 20 µL washing drops before being moved to a new, fresh individual 10 µL drop until day 6. On day 6, the blastocyst was removed from the culture drop to perform the TE biopsy and 8–9 µL of SBM was collected. The TE biopsies were loaded into a 0.2 mL PCR tube prefilled with 2.5–3 µL of loading buffer, whereas the SBM samples were loaded into an empty 0.2 mL PCR tube. The samples were stored at −20 °C for at least 24 h before shipping them to the genetics laboratory. After the TE biopsy, the blastocysts underwent the vitrification procedures routinely used by each IVF center. A negative control sample from each patient, consisting of a 10 µL drop of medium processed under the same culture and collection protocol but without embryo exposure, was also collected and analyzed.

After obtaining the TE result, a series of ICM biopsies were performed in 290 warmed blastocysts donated for research. The samples were loaded into 0.2 mL PCR tubes prefilled with 2.5–3 µL of loading buffer and stored at −20 °C for at least 24 h before shipping to the genetics laboratory.

**Figure 2:**
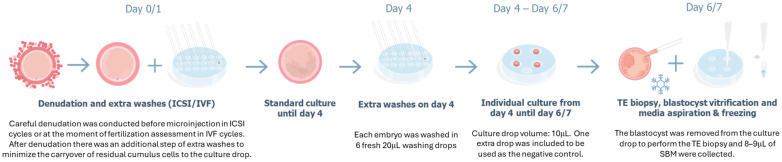
Protocol for niPGT-A.

To prevent external contamination of the SBM samples, research personnel were adequately trained and wore a mask, cap, long-sleeved gown, and gloves while handling the embryos; additionally, the IVF laboratory materials used were dedicated exclusively to the study in a clean area. To minimize maternal contamination, careful denudation of surrounding cumulus cells was performed before microinjection in ICSI cycles or at the time of fertilization assessment in IVF cycles, with an additional step of extra washes to minimize the carryover of residual cumulus cells into the culture drop. The embryos did not undergo any additional manipulation, such as assisted hatching or collapse during the culture period before biopsy (see the detailed protocol in Figure 2).

Blastocysts were graded for blastocyst expansion, and for the quality of the ICM and TE using the Gardner classification criteria [23].

### 2.6. Sample Analysis

Whole-genome amplification (WGA) and DNA barcoding were performed on DNA from the TE and ICM biopsies and the SBM samples using the Ion ReproSeq PGS Kit (Thermo Fisher Scientific, Waltham, MA, USA), with a modified protocol for SBM due to the specific characteristics of embryonic cfDNA. Successful WGA was evaluated by loading 1.5 µL of amplified products on an agarose gel (Lonza, Basel, Switzerland). Samples were pooled in batches of 24 or 96 samples. Libraries were quantified using the Qubit High Sensitivity dsDNA Kit (Life Technologies, Waltham, MA, USA), diluted and loaded onto the Ion Chef (Thermo Fisher Scientific, Waltham, MA, USA) for automated template preparation and chip loading. For the TE and ICM biopsies, the standard next-generation sequencing (NGS) protocol for 96 samples (a 530 chip) was used. For SBM, runs of 24 and 96 samples were performed (520 and 530 chips). Sequencing of the chips was carried out using the S5 XL sequencer (Thermo Fisher Scientific, Waltham, MA, USA).

### 2.7. Interpretation of Sequencing Data and Diagnosis

The sequencing data were processed and automatically uploaded to the Ion Reporter Software (Thermo Fisher Scientific, Waltham, MA, USA). For all samples, data obtained from the BAM files for each embryo were analyzed using a proprietary algorithm. The samples were categorized as informative (euploid or aneuploid) or non-informative (in the case of samples that could not produce an interpretable result). Non-informative results were sub-categorized into non-informative quality control (QC) (when they did not reach minimal sequencing quality parameters, i.e., poor quality profiles), chaotic (when 6 or more whole chromosome aneuploidies were present), and no DNA detected (in cases where not enough cfDNA was present and thus was not able to sufficiently amplify and produce a result). For informative TE and ICM biopsies, whole-chromosome aneuploidies, mosaicism (between 30–70%), and segmental aneuploidies (deletion or duplication > 10 Mb) were identified. For informative SBM samples, only two categories were established, euploid and aneuploid (whole-chromosome aneuploidies and segmental aneuploidies > 10 Mb), according to the difference value (DV) observed for each chromosome with the expected 2 copy numbers. DV thresholds had been previously established according to the results obtained in the interim analysis of this study [21]. Based on that data, it was observed that the best sensitivity and specificity were obtained when considering DVs of approximately 50% for most chromosomes.

### 2.8. Clinical Outcomes

**Figure 3:**
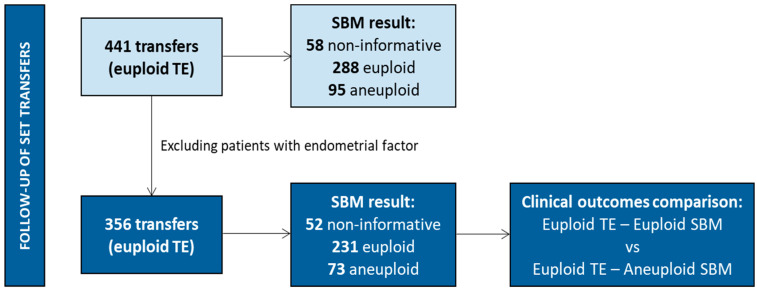
Clinical outcome follow-up. SETs performed. Only frozen single embryo transfers of euploid blastocysts (based on the result of the invasive TE biopsy) were performed. The results of the corresponding SBM samples for those blastocysts are detailed.

During the study, 441 frozen single embryo transfers (SET) of a euploid blastocyst (based on the result of the invasive TE biopsy) were performed. To properly analyze the embryo’s contribution to reproductive success, patients with identified endometrial factor or patients with previous endometrial receptivity and/or microbiome tests, were excluded for further comparisons. Clinical outcomes of the embryos transferred were classified into two groups: when the TE and SBM from the same embryo were euploid and concordant, and when a euploid TE and an aneuploid SBM were observed for the same embryo (Figure 3). No mosaic embryos were transferred based on PGT-A results.

The warming procedures prior to transfer and the endometrial preparation were performed according to each clinic’s standard of care. Follow-up for all SETs was performed until the time of delivery or miscarriage. Biochemical pregnancy loss was defined as positive serum β-hCG levels in at least two pregnancy tests but without any ultrasound evidence of pregnancy. A clinical pregnancy was defined as the presence of a gestational sac with fetal heartbeat. A clinical miscarriage was defined as a pregnancy loss after previous confirmation of a gestational sac by ultrasound. The rate of positive pregnancy tests, clinical pregnancy rate, and live birth rate were calculated based on the number of vitrified–warmed embryo transfers performed. The rate of clinical miscarriage was calculated based on the number of clinical pregnancies.

### 2.9. Statistical Analysis

Informativity rates (the number of samples with interpretable results according to quality parameters divided by total number of samples analyzed) were estimated individually for the TE and ICM biopsies and the SBM samples. Concordance rates between sample types were calculated only when all the samples included in the comparison yielded informative results. The ploidy concordance rate was defined as a matching result (both euploid or both aneuploid). Ploidy concordance was divided into two subcategories: total concordance (chromosomal status for all chromosomes in both samples was the same, regardless of the type of aneuploidy) and partial concordance (chromosomal status for some chromosomes might differ between the samples, but both samples were aneuploid). Concordance was calculated as the number of concordant samples divided by the total number of informative samples.

False positive (gold standard sample is euploid, but comparison sample is aneuploid) and false negative (gold standard sample is aneuploid, but comparison sample is euploid) rates, specificity (frequency of true negative results), sensitivity (frequency of true positive results), positive predictive value (likelihood of true positives), and negative predictive value (likelihood of true negatives) were determined. For the SMB–TE, SBM–ICM, and TE–ICM comparisons, the gold standard samples are TE, ICM, and ICM, respectively. Data is presented globally and by individual center.

Descriptive data for continuous variables are presented as mean and standard deviation, and categorical data as counts and percentage. For continuous variables, either the independent sample Student’s t-tests or the Wilcoxon rank sum tests were performed. For comparing categorical data, Chi-squared tests were performed and non-parametric Fisher exact tests were applied when the sample sizes were small. Nominal variables were analyzed using the Cochran–Armitage test to detect trends. Statistical significance was set at *p* < 0.05. Univariate logistic regression was performed for specific variables. Multivariate logistic regression was applied to assess the impact of patient characteristics, ovarian stimulation, embryo culture, and blastocyst quality on SBM informativity rate and SMB–TE ploidy concordance rate. Statistical analyses were performed using R 4.5.1 (13 June 2025).

## 3. Results

### 3.1. Patient Demographics and Cycle Characteristics

In summary, 850 cycles from 716 patients were included from the 10 participating clinics. Most patients (n = 604) had one cycle, although this number increased to two, three, four, or even six, in some cases. The female mean age was 36.8 years, with a standard deviation of 4.9. Advanced maternal age (AMA) was the primary PGT-A indication in 65.1% of the cycles. The majority of cycles were performed with autologous oocytes (only 8.9% were performed with donated oocytes) (Supplemental Table S3) or autologous sperm (only 4.8% with donated sperm). In addition, ICSI represented the predominant insemination technique (89.8%) (Supplemental Table S4). As expected, a decrease in the number of embryos, i.e., the SBM samples available, as well as an increase in the incidence of aneuploidies was observed with increasing female age, reaching statistical significance in both cases (*p*-value < 0.001) (Supplemental Table S5).

Supplemental Tables S3–S5 provide a detailed description of the patients’ demographic characteristics, their clinical background and cycle characteristics.

### 3.2. Blastocyst Quality

From the 2539 blastocysts included in the study, 91% (n = 2310) were day 6 blastocysts and only 9% (n = 229) were day 7 blastocysts. Regardless of the day of development, most blastocysts were expanded (75.3%) and showed good quality (A, B) for both ICM and TE: 77.6%. Only 7.1% of the blastocysts were fully hatched (Table 1).

### 3.3. SBM Informativity and Concordance vs. TE Biopsies

For each of the 2539 blastocysts included in the study, a TE biopsy and the corresponding SBM sample were analyzed (Figure 1). The overall informativity rate for the cfDNA present in SBM was 88.7% with 4.0% of samples classified as chaotic, 5.3% as non-informative and 2.1% as no DNA detected. Regarding the TE biopsies, the informativity rate increased to 96.8%, with 1.1% of chaotic samples, 1.2% of non-informative and 0.9% with no DNA detected.

To compare the chromosome copy number results between the embryonic cfDNA and the TE biopsy, stratification was applied according to the Standards for Reporting of Diagnostic Accuracy Studies criteria (Supplemental Figure S1), where the index test was the SBM sample analyzed and the reference standard was the TE biopsy.

**Figure 4:**
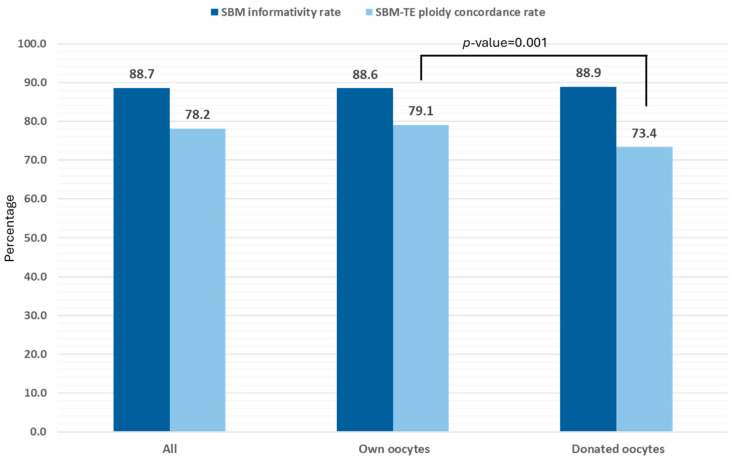
SBM informativity rate and SMB–TE ploidy concordance rate for the blastocysts analyzed in the study. The values were calculated as overall values and depending on the oocyte’s origin. Of the 2539 blastocysts included, 2105 were derived from cycles using the patients’ own oocytes, whereas 434 were derived from ovum donation. *p*-values shown when statistically significant differences were observed.

The two most relevant parameters on the niPGT-A studies, the SBM informativity rate and the concordance rate (in our case, the SBM–TE ploidy concordance rate), were assessed depending on oocyte origin (autologous vs. donor). From the 2539 blastocysts included in the study, 2105 were derived from cycles that used the patients’ own oocytes, whereas 434 were derived from oocyte donation. As depicted in Figure 4, the SBM informativity rate was similar, independent of oocyte origin (88.6% for own vs. 88.9% for donated oocytes; *p*-value = 0.9). However, the SBM–TE ploidy concordance rate was statistically different: 79.1% for own vs. 73.4% for donated oocytes (*p*-value = 0.001). It is noteworthy that cycles with donated oocytes were present in only four of the 10 IVF centers participating in the study. Additionally, the distribution of ovum donation cycles among these centers was unbalanced, with almost 75% of the samples, and therefore the results, coming from one single center, thus reducing the representativeness of the information obtained about niPGT-A performance in this type of cycles. For these reasons, the subsequent detailed analysis of the results was based only on embryos derived from cycles using the patients’ own oocytes.

### 3.4. SMB–TE Ploidy Concordance Rate (Cycles with Patients’ Own Oocytes)

The overall ploidy concordance for the 1832 SMB–TE pairs with informative results in patients using their own oocytes was 79.1% (range: 74.1–82.1). The overall ploidy concordance rate was composed of 64.8% of total concordance plus 14.3% of partial concordance. In addition, 1.4% of the pairs showed euploid status for both the SBM sample and TE biopsy but with different sex chromosomes. Regarding the discordances, the overall false positive rate was 10.7% and the overall false negative rate 8.8%. We speculate that, based on the comparison of the NGS profiles from both the SBM samples and TE biopsies, where differences were observed for sex and/or DVs for sex and autosomal chromosomes, maternal cumulus cell contamination (7.5%), external contamination (0.6%) or polar body contamination (0.3%) in the SBM samples account for the majority of the false negatives observed. Other parameters such as specificity, sensitivity, positive predictive value (PPV) and negative predictive value (NPV) were calculated to determine the overall performance of the test. The overall results obtained were 77.3%, 82.9%, 80.1% and 80.4%, respectively, with some clinics achieving values closer to, and even exceeding, 90% for specific parameters, highlighting the capacity of the test to correctly identify euploid and aneuploid embryos when following the modified embryo culture protocol required for niPGT-A (Table 2).

When considering the concordance per chromosome, it ranged between 95.1% and 97.8% for autosomes. For sex chromosomes, it was 92.5% for SBM samples reported as XX, and 98.8% for SBM samples reported as XY.

### 3.5. Factors Influencing SBM Informativity Rate and SMB–TE Ploidy Concordance Rate

A univariant analysis was performed to assess the influence of four key variables on SBM informativity rate and SMB–TE ploidy concordance rate: center (Table 2), female age (Supplemental Figure S2), embryo expansion degree and ICM/TE quality (Supplemental Figure S3), and culture conditions (incubator model and medium brand) (Supplemental Figure S4).

Regarding the SBM informativity rate, there were significant differences when considering the culture conditions. Specifically, the SBM informativity rate was different depending on the incubator model (*p*-value < 0.001) and the medium brand (*p*-value = 0.006). The same observation was obtained when considering the influence of the center, finding differences in the SBM informativity rate among the 10 participating IVF centers (range of 84.8–95.1%; *p*-value = 0.04). Therefore, the observed differences in the SBM informativity rate could be due to the culture conditions but also to the participating centers.

Regarding the SMB–TE ploidy concordance rate, there were statistically significant results only when considering the quality of TE: it was observed that poorer TE qualities were correlated with higher SMB–TE ploidy concordance rates (*p*-value = 0.02).

These significant differences disappeared when a multivariate analysis was performed, including variables related to patient characteristics, ovarian stimulation, embryo culture, and blastocyst quality. For the SBM informativity rate, the influence of the center and the incubator/medium used did not show statistical significance, although the impact of the clinic showed a trend towards significance (*p*-value = 0.06 vs. *p*-value = 0.24). However, female body mass index (adjusted odds ratio = 1.08; 95% confidence interval = 1.03–1.13, *p*-value = 0.003) and number of metaphase II oocytes (adjusted odds ratio = 1.13; 95% confidence interval = 1.03–1.25, *p*-value = 0.01) represented the only parameters with statistically significant results, both directly correlating to the SBM informativity rate. Whereas for the SMB–TE ploidy concordance rate, only the number of NGS reads obtained in SBM sequencing, i.e., the quality of the sample, directly correlated to the SMB–TE ploidy concordance rate (adjusted odds ratio = 1.19; 95% confidence interval = 1.01–1.41, *p*-value = 0.04).

### 3.6. Incidence of Aneuploidies

**Figure 5:**
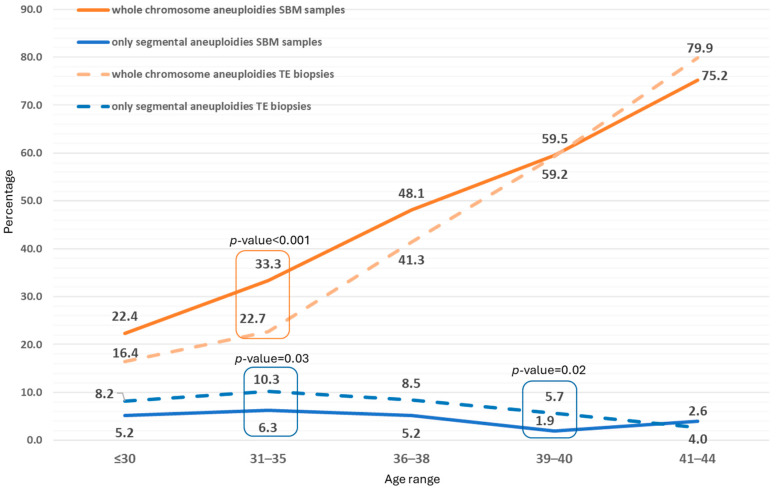
Incidence of aneuploidies in each age range in SBM samples and TE biopsies. Two categories were considered: presence of whole chromosome aneuploidies or only segmental aneuploidies. *p*-values shown when statistically significant differences were observed.

In the 1832 informative SBM samples from blastocysts derived from the patients’ own oocytes, 48.9% showed whole chromosome aneuploidies, 4.6% showed only segmental aneuploidies, and 46.5% showed euploid results. The incidence of aneuploidies according to female age followed a similar pattern in the TE biopsies and SBM samples when considering whole chromosome aneuploidies and only segmental aneuploidies, although some differences were observed in specific age groups (Figure 5).

The mean number of aneuploidies in the cfDNA samples and in the TE biopsies in the samples with abnormal results was 1.864 (standard deviation: 0.97) and 1.53 (standard deviation: 0.96), respectively. Regarding the incidence of aneuploidies per chromosome, the higher incidence observed in the SBM and TE biopsies was for chromosomes 15, 16, 21, and 22 with values above 5% (Supplemental Figure S5).

### 3.7. Contribution of the ICM

In two of the 10 participating IVF centers, the approved study protocol also included the analysis of ICM biopsies from donated blastocysts. Therefore, it was possible to conduct an additional analysis in the 290 day 6 and day 7 embryos donated for research (Supplemental Table S1). The SBM samples, TE biopsies, and ICM biopsies were analyzed (Figure 1). In total, the three sample types (SBM, ICM and TE) were informative in 230 of the blastocysts analyzed. Considering the ICM biopsy as the reference, the ploidy concordance for the SBM samples was 87.0% (200/230) and for the TE biopsies was 90.0% (207/230), without statistically significant differences. The false positive rates were similar for the SBM samples and TE biopsies (5.2% and 7.8%, respectively), and false negative rates, mainly due to potential contamination with maternal DNA, were higher in the SBM samples (7.8%) than in the TE biopsies (2.2%) without statistically significant differences. The ploidy concordance between the TE biopsies and the embryo cfDNA samples was 89.1% (205/230) (Table 3). When considering the average concordance rate per chromosome for the three comparisons (SBM–ICM, TE–ICM, and SMB–TE), the respective values were 95.6%, 97.3%, and 96.0%.

When focusing on the prevalence of segmental aneuploidies in the 230 SBM samples, they were present in only 31 SBM: in 14 SBM without additional whole chromosome aneuploidies, and in 17 SBM in combination with whole chromosome aneuploidies, following the expected prevalence already presented in Figure 5. The ploidy concordance rates when compared to ICM and TE were 90.3%, and 93.6%, respectively.

A univariate logistic regression analysis revealed that female age positively correlated with the SBM–ICM (odds ratio = 1.10, 95% confidence interval = 1.01–1.20) and the TE–ICM ploidy concordance rates (odds ratio = 1.26, 95% confidence interval = 1.15–1.39). No correlation was found for the SMB–TE comparison. When the results were stratified by blastocyst expansion degree (expanded, hatching, or fully hatched) or embryo quality no differences were observed.

### 3.8. Clinical Outcomes

To analyze the impact of SBM results on clinical outcomes, we focused on patients without endometrial factor and transfers from cycles performed with the patients’ own oocytes. Therefore, from the initial 441 SETs performed, only 309 were considered for analysis. In all cases, euploid blastocysts, determined by the TE biopsy, were transferred. The respective SBM sample was non-informative in 14.2% of the cases (44/309). Regarding informative media samples, the SBM was also euploid in 65.7% (203/309) and aneuploid in 20.1% (62/309) of the cases. Despite the differences observed in concordance, both groups presented similar characteristics. Female age (euploid TE–euploid SBM and euploid TE–aneuploid SBM) was similar (36.8 years vs. 36.1 years), as well as the percentage of cases with ICSI or IVF (ICSI: 89.7%; IVF: 10.3% in euploid TE–euploid SBM; and ICSI: 85.5%; IVF: 14.5% in euploid TE–aneuploid SBM), and blastocyst quality (good: 85.7% vs. 83.9%; poor: 14.3% vs. 16.1%, respectively).

**Figure 6:**
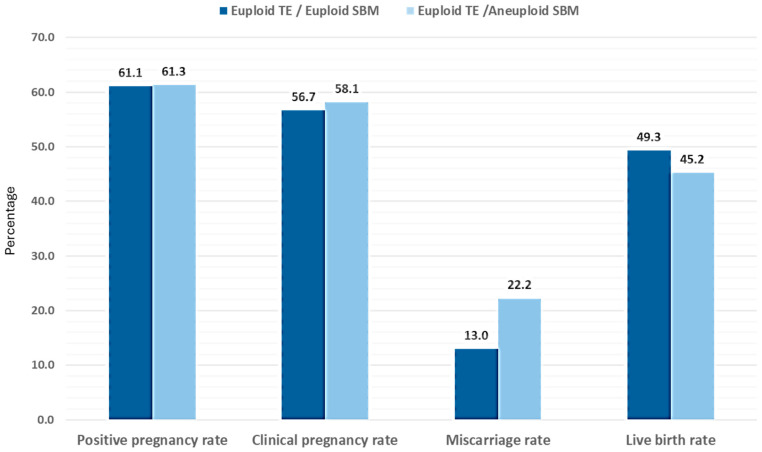
Clinical outcomes obtained for the SETs performed. The positive pregnancy rate, clinical pregnancy rate, and live birth rate were calculated based on the number of vitrified–warmed embryo transfers performed. The rate of clinical miscarriage was calculated based on the number of clinical pregnancies. 203 SETs were included in the euploid–euploid group (mean age: 36.8 years ± 3.5) and 62 SETs were included in the euploid–aneuploid group (mean age: 36.1 years ± 3.5).

In addition, no significant differences were observed in pregnancy rates. We observed a trend towards lower miscarriage rate when the TE biopsy and the SBM sample were both euploid and concordant (13.0%) compared to when the TE biopsy was euploid and the SBM sample was aneuploid and thus discordant (22.2%), thus resulting in a trend towards higher live birth rates with euploid TE–euploid SBM (Figure 6). A formal post-hoc power analysis was not conducted; nevertheless, the small sample size indicates that the study may lack sufficient statistical power to identify moderate differences in miscarriage rates. Although the clinical outcome information presented here is based on a specific subgroup of cases, Supplemental Table S6 provides detailed information of the clinical outcomes observed for all 441 transfers as well as those from patients without endometrial factor and from cycles with ovum donation. In all cases, the same trend for the miscarriage rate was observed. In addition, the information from the euploid TE–non-informative SBM group is also presented. Interestingly, euploid TE–non-informative SBM embryos showed similar miscarriage rates than euploid TE—euploid SBM embryos and, thus, lower to euploid TE—aneuploid SBM embryos.

## 4. Discussion

niPGT-A has recently emerged as a prioritization tool for embryo selection. This technique analyzes the cfDNA released by the embryo into the drop of culture medium where it has been growing up to the blastocyst stage. Based on the results obtained, the embryos can be ranked from first to last for transfer.

However, certain key aspects of niPGT-A remain under debate. The most contentious aspect relates to the value of embryonic cfDNA present in the SBM and therefore the representativeness of the information provided by the test.

From a technical perspective, researchers have worked diligently for the last decade to establish the optimal embryo culture and media analysis protocols to improve the results that can be obtained from SBM. Despite the distinct strategies applied in these studies, several key points have been identified as crucial to the success of the technique [19,20]. These key points include controlling contamination to minimize the presence of external and/or maternal DNA in the SBM [24], collecting the SBM on day 6 (and not on day 5) of embryo development to optimize the amount and quality of the embryonic cfDNA [12,13,15,25], and adapting cfDNA amplification and sequencing methods as well as developing customized algorithms to analyze results [10,11].

An adapted embryo culture protocol must be followed in the IVF laboratory to ensure the success of the niPGT-A. However, any modification to the standard culture process can raise concerns in the IVF centers. Controlling contamination is the first step. External contamination can be controlled with routine clean laboratory measures. This implies working in a clean area that is, where possible, dedicated to the niPGT-A protocol. The donning of a face mask, cap, and long-sleeved gown are also mandatory. To avoid maternal contamination from cumulus cells, carefully washing both oocytes and embryos after denudation, and including extra washes on day 4 of development remains a basic requirement. The second change involves decreasing the culture drop volume from day 4 (after the extra washes) until the time of media collection. The embryo must be cultured in a reduced volume to allow the subsequent analysis of the SBM by currently developed WGA protocols. Published studies have not reported negative impacts on embryo quality due to the decrease in the culture drop volume [26,27]. However, it is the third change in the routine laboratory procedure that raises the main concern about embryo quality and survival. That is extending embryo culture for media collection and blastocyst vitrification from day 5 to day 6 of development, even if the embryo has a suitable quality of blastocyst stage on day 5. Encouragingly, Sakkas et al., 2024, reported that fixing the time of vitrification for all embryos to day 6 did not adversely affect embryo viability and reproductive potential [22]. Moreover, our interim analysis [21] revealed that the majority of the 1301 blastocysts analyzed were expanded and had good quality (quality A or B based on Garner’s criteria for both embryonic compartments) on day 6. The same results have been confirmed with the global dataset of the study presented in the current manuscript, with almost double the number of embryos analyzed (n = 2539): 78.6% of blastocysts had good quality on day 6, 75.3% were expanded and only 6.9% were fully hatched. In addition, good clinical outcomes have been obtained from the transfers performed, especially for the clinical pregnancy rate and live birth rate.

Regarding the analysis of the SBM, two parameters are used to determine niPGT-A success: SBM informativity and the concordance with other embryonic samples (mostly a TE biopsy as PGT-A represents the gold standard for aneuploidy detection). Adapting the SBM amplification method, for example, by skipping the lysis step [28], and developing proprietary algorithms with specific mosaicism thresholds to analyze the sequencing results [10,11] are crucial for optimizing both values. In an interim analysis of this multicenter study [21], we previously reported an SBM informativity rate and SMB–TE ploidy concordance rate of approximately 90% and 80%, respectively. The current study expands on these results, reporting an SBM informativity rate of 88.7% (chaotic results considered as non-informative) and an average SMB–TE ploidy concordance rate value of 79.1% (range between 74.1% and 82.1%). When considering the concordance per chromosome, it ranged between 95.1% and 97.8% for autosomes. For sex chromosomes it was 92.5% for SBM samples reported as XX, and 98.8% for SBM samples reported as XY. In addition, the values for other statistical parameters (such as sensitivity, specificity, PPV, and NPV) remained above 80% in many cases. Of note, in the sub-analysis performed in the subset of blastocysts in which an ICM biopsy was also performed and considered the reference value, the concordance rate for the SBM–ICM comparison was 87.0%, very close to the TE–ICM concordance rate of 90.0%. Moreover, the concordance rate for the SMB–TE comparison in this subset of samples was 89.1%. All this data was obtained for cycles with autologous oocytes. When comparing our results with other related studies, the values obtained fell within the range of those previously published for fresh cycles, being 99.3% for the SBM informativity rate and 87.5% for the SMB–TE ploidy concordance rate, representing the highest values obtained so far [14]. These values are slightly higher when analyzing previously vitrified embryos, reaching 100% for the SBM informativity rate [29], and 89.1 and 93.8% for the SMB–TE and SBM-WB concordance rates, respectively [10,30].

It is essential to emphasize that, although the ploidy concordance rate values obtained are high, they imply that approximately one in five embryos may present discordant results between SBM and TE biopsy. Therefore, when using SBM results alone for clinical decision-making, they need to be interpreted accordingly: as a prioritization tool but not as a diagnostic test. Further improvements in sensitivity and specificity are necessary in order to consider niPGT-A a diagnostic test rather than a prioritization tool. In addition, a better understanding of partial concordances—from both a technical and a clinical perspective—will also help to improve niPGT-A performance.

Cycles with donated oocytes were also included in the study. However, there was substantial variation in both the number of centers that participated and the number of cycles contributed by each center, resulting in a markedly skewed distribution with most samples originating from a single center. Although the SBM informativity rate was not different for cycles with donated and autologous oocytes, the lower overall ploidy concordance rate observed and the values obtained for other parameters such as sensitivity and specificity were affected due to this uneven sample distribution, therefore making it difficult to draw definitive conclusions about niPGT-A performance in this type of cycles.

When evaluating potential factors that may have impacted both parameters in our study, it was observed that, in the univariant analysis, the culture conditions (media and incubator used for embryo culture) and the center were significantly associated with the SBM informativity rate obtained. However, these significant differences disappeared in the multivariate analysis which also included variables related to patient characteristics, ovarian stimulation, embryo culture, and blastocyst quality.

To date, published studies have not identified any specific culture medium or incubator that offer superior performance for niPGT-A [21]. The same conclusion has been obtained in this study. Although plenty of culture condition set ups, i.e., combinations of incubators and media, have been already analyzed without finding any significant effect on niPGT-A performance, further research on this aspect, as well as on possible improvements on culture conditions to optimize cfDNA release, would be interesting.

Regarding the effect of the center, the values obtained fall within the range of those previously published showing some inter-center variability due to, for example, differences in laboratory context and stochastic technical factors, causing small differences in niPGT-A informativity and concordance. The embryo culture protocol presented here is thus robust and guarantees the success of niPGT-A as a tool for embryo transfer prioritization. Nevertheless, it is important to remark on this aspect: that niPGT-A protocol adherence in the IVF center is key to obtaining optimal results. Therefore, it is essential that any IVF center interested in implementing niPGT-A establishes a quality control program, as with any other technique performed in the clinic. The program must consist of, initially, an on-site niPGT-A validation to confirm that the required embryo culture protocol modifications do not negatively impact embryo quality and to verify whether the SBM informativity rate and ploidy concordance values, as well as the percentage of contamination observed in the negative controls, reach the minimum required thresholds, demonstrating that the center has completed the necessary learning curve and can proceed with clinical cases. Considering the values obtained in this study, a validation could be considered as “passed” when the SBM informativity rate reaches around 90% or above, the concordance for the SBM result with other embryonic sample reaches at least 80%, and the negative controls contain no contamination (or almost none). It should be noted that even higher standards could be established considering the validation results obtained by a group of clinics interested in implementing niPGT-A that followed the embryo culture protocol proposed in this study [31]. Once the center has passed the validation and is already performing niPGT-A clinical cases, whose transfers will be based only on the result of the SBM, periodically checking key performance indicators (KPIs) to ensure that the quality of the samples, and thus the results, remain unaffected should be mandatory.

From a biological point of view, the origin of the embryonic cfDNA present in the SBM remains unknown. Although there is no doubt about the fact that there is DNA in the drop of culture medium and about its embryonic origin [6,7,18], it is debatable whether that cfDNA originates from the TE, the ICM or both compartments in an undefined proportion. Chen et al., 2021 concluded that the embryonic cfDNA present in the SBM originates one-third from TE and two-thirds from ICM [32]. In our study we observed similar high concordance rates between the three embryonic compartments (SBM–ICM: 87.0%; SMB–TE: 89.1%; TE–ICM: 90.0%). These values are even higher than those reported in the interim analysis of the study [21] and demonstrate the high representativeness that the information provided by the SBM has on the actual genetic composition of the blastocyst.

Despite these promising values, whether the embryonic cfDNA constitutes a better sample for determining the aneuploidy status of the blastocyst than the few trophectoderm cells obtained by a biopsy remains uncertain. The use of cfDNA would avoid current concerns regarding the invasive procedure (including mosaicism), although we require more studies to determine the precise origin of cfDNA and how it is released. Two main hypotheses have been proposed so far to explain potential mechanisms. The first mechanism links cfDNA and embryonic self-correction, assuming that the genetic material present in the SBM derives from aneuploid cells that entered apoptosis [33,34,35]. However, it has been observed that all embryos release cfDNA (euploids and aneuploids) and they do it in similar amounts [18]. This led to another hypothesis, suggesting that cfDNA release could reflect a constitutive mechanism during embryonic development, possibly due to cellular and/or nuclear reorganizations [36]. Factors such as the function of this genetic material, if any, and its characteristics (length and structure) remain poorly explored.

Another key aspect of the debate concerns the clinical benefit of niPGT-A. Several groups have evaluated clinical outcomes when transferring euploid embryos based on SBM analysis. Xi et al. conducted a retrospective cohort study on 273 patients with recurrent implantation failure (RIF) or recurrent pregnancy loss (RPL). Patients receiving euploid embryo transfers identified by niPGT-A (study group) achieved superior clinical results compared to those selected only by morphology (control group). This study reported a clinical pregnancy rate of 46.9% in the study group in comparison to 28.7% in the control group for RIF patients, and an ongoing pregnancy rate of 40.7% in the study group vs. 25.0% in the control group for RPL patients [37]. Chen et al. and Nakhuda et al. evaluated the pregnancy outcomes in 212 and 120 SETs, respectively. Both studies demonstrated better results when the embryo, transferred based on morphological assessment, was euploid: the live birth rate was 46.1% vs. 22.1% and the clinical pregnancy rate was 64.0% vs. 44.4% for euploid and aneuploid embryos, respectively [38,39]. Most recently, Sun et al. conducted a three-arm study comparing clinical outcomes for transfers selected by morphological assessment, niPGT-A, and conventional PGT-A. Findings showed that using either niPGT-A or conventional PGT-A to select euploid embryos raises the cumulative live birth rate in women aged 35–40 versus morphology-based selection [40]. Combined, these data suggest that niPGT-A can improve clinical outcomes in a similar manner to conventional PGT-A and can add benefit to the use of stand-alone morphology-based grading. However, more clinical data is needed and thus, for now, niPGT-A should be used as a tool for embryo prioritization and not as a diagnostic tool. Euploid embryos should be considered first for transfer, followed by other embryos with lower scores (depending on the number and type of abnormalities observed—whole chromosome or segmental aneuploidies). The concordance patterns for whole chromosome aneuploidies, segmental aneuploidies and combinations of both might differ, highlighting the necessity to properly establish embryo rankings that prioritize the embryos with the highest probability of being euploid.

In our study, all 265 embryos were transferred according to the TE result. Although in all cases the PGT-A result was euploid, we observed a lower miscarriage rate (13.0%) when the corresponding SBM result was also euploid, compared to when the SBM was discordant and aneuploid (22.2%). No statistical significance was achieved. The same trend was obtained in one of our previous publications, but in a smaller sample size (n = 29) [12]. These findings might highlight the relevance of the information provided by the embryonic cfDNA present in the SBM, although more studies, preferentially non-selection with embryo transfers only based on SBM results, are needed to directly evaluate niPGT-A’s potential to improve clinical outcomes.

In summary, this study, performed with the highest sample size analyzed to date, underscores niPGT-A as a promising non-invasive technique for aneuploidy detection. The embryo culture protocol used has been confirmed to be robust across different laboratories without affecting embryo viability and potential. In addition, high informativity and concordance rates have been obtained and relevant information about the origin of the cfDNA has been provided. However, despite its great potential there are some limitations related to niPGT-A that need to be mentioned.

On one hand, the embryo culture protocol that needs to be followed for the success of the technique entails slight modifications of the standard routine and, although no detrimental effect for the blastocysts has been described, any changes raise concerns for IVF clinics. Therefore, to increase the implementation of niPGT-A, more research should be focused on minimizing those changes. More precisely, studies may seek alternatives to allow media collection also on day 5 and avoid extra washes. Currently, the accuracy of the results would be seriously affected by the low amount and quality of the embryonic cfDNA present in the sample if performing this alternative protocol. Understanding embryo development and its correlation with cfDNA release and incorporating microfluidics into culture devices could benefit and greatly improve niPGT-A. Thus, studies including morphokinetics and automation will be beneficial to decipher if it is possible to maintain niPGT-A success while avoiding what can be considered as disturbances during blastocyst development.

On the other hand, it is necessary to optimize cfDNA analysis to increase specificity and sensitivity to even higher levels and also develop methods to detect the contamination present in SBM samples. Although following the specific niPGT-A culture protocol, which establishes the need to work under clean conditions and includes negative controls, minimizes the risk of contaminating SBM samples, new molecular methods for analyzing samples and bioinformatic algorithms to detect, quantify and subtract contamination levels are necessary [41,42] as currently only the analysis of NGS profiles, mostly for XY SBM samples, is available. Moreover, widening the use of SBM from aneuploidy detection to ploidy, translocations and monogenic diseases should be explored, if contamination can be accurately identified.

We consider niPGT-A a potentially clinically relevant tool for embryo prioritization, especially useful for patients with no PGT-A indications, patients with previously vitrified embryos [43] who have had unsuccessful previous transfers, and couples who prefer not to biopsy their embryos or do not have access to invasive PGT-A. niPGT-A can also be easily applied to embryos with slightly lower blastocyst grades, including those with C-grade TE or ICM. Selection of these embryos remains a significant challenge and many are currently discarded even though they have potential to lead to a live birth [44]. By combining the technical improvements related to embryo culture and sample analysis as well as by collecting more information regarding the clinical benefit according to specific age groups and indications, the current use of niPGT-A as a prioritization tool [45,46,47] could be further evaluated. This prioritization technique is superior to morphology, but we require additional studies to determine first whether niPGT-A can reach the status of the invasive PGT-A and, if so, replace it.

## Figures and Tables

**Table 1:** Blastocyst quality.

	Day 6	Day 7	Total
**No. of blastocysts (%)**	2310 (91.0)	229 (9.0)	2539 (100)
**Expansion degree (%):**			
**Early blastocyst**	10/2308 (0.4)	0	10/2537 (0.4)
**Cavitated blastocyst**	70/2308 (3.0)	7/229 (3.1)	77/2537 (3.0)
**Expanded blastocyst**	1737/2308 (75.3)	172/229 (75.1)	1909/2537 (75.3)
**Hatching blastocyst**	331/2308 (14.3)	28/229 (12.2)	359/2537 (14.2)
**Fully hatched blastocyst**	160/2308 (6.9)	22/229 (9.6)	182/2537 (7.1)
**ICM quality (%):**			
**A**	883/2308 (38.3)	38/228 (16.7)	921/2536 (36.3)
**B**	1077/2308 (46.7)	141/228 (61.8)	1218/2536 (48.0)
**C**	348/2308 (15.1)	49/228 (21.5)	397/2536 (15.7)
**TE quality (%):**			
**A**	1001/2308 (43.4)	33/228 (14.5)	1034/2536 (40.8)
**B**	968/2308 (41.9)	133/228 (58.3)	1101/2536 (43.1)
**C**	339/2308 (14.7)	62/228 (27.2)	401/2536 (15.8)
**Good quality blastocysts (%)**	1815/2308 (78.6)	152/228 (66.7)	1967/2536 (77.6)

Blastocyst classification following Gardner’s criteria [23]. Values are presented as n/N (%). Percentages were calculated considering the number of embryos with informative data for each variable. Blastocysts with ICM and TE quality with grades A or B were categorized as good quality embryos (AA, AB, BA, and BB), whereas the remaining embryos were categorized as poor quality embryos (AC, CA, BC, CB, CC).

**Table 2:** SBM informativity, ploidy concordance, specificity, sensitivity, PPV, and NPV for the 10 participating IVF centers (cycles with own oocytes). As both PPV and NPV are prevalence dependent, the aneuploidy prevalence (=the percentage of aneuploid TE biopsies; TE biopsy is the reference for aneuploidy detection) has been used to adjust them. Therefore, that information has also been included in the table.

IVF Center	A	B	C	D	E	F	G	H	I	J	Overall	95% Confidence Interval
**No. of Informative SMB–TE Pairs**	50	315	102	337	18	152	469	162	54	173	1832	
**SBM Informativity**	87.7	91.9	90.2	87.0	94.7	95.1	87.1	87.2	90.2	84.8	88.6	
**Ploidy Concordance**	80.0	81.0	80.4	74.8	77.8	81.6	80.2	82.1	74.1	77.5	79.1	
**Specificity**	77.8	80.1	82.1	71.5	83.3	80.9	71.9	88.3	68.4	75.8	77.3	(74.4–80.0)
**Sensitivity**	90.5	84.6	83.7	78.5	81.8	84.2	87.1	79.3	77.1	80.2	82.9	(80.4–85.2)
**PPV**	74.7	74.9	77.3	81.8	88.5	72.6	82.0	87.4	81.8	74.5	79.7	
**NPV**	91.9	88.1	87.4	67.1	74.4	89.5	79.1	80.6	61.9	81.3	80.8	
**Aneuploidy Prevalence**	42.0	41.3	42.2	62.0	61.1	37.5	59.5	50.6	64.8	46.8	51.7	

Values are presented as n (for the informative pairs) or % (for the remaining parameters). Values above 80.0 are highlighted.

**Table 3:** Concordance rates between SBM samples, TE biopsies and ICM biopsies.

	Ploidy Concordance	Total Concordance	Partial Concordance
**SMB–TE**	205/230 (89.1)	142/230 (61.7)	63/230 (27.4)
**SBM–ICM**	200/230 (87.0)	130/230 (56.5)	70/230 (30.4)
**TE–ICM**	207/230 (90.0)	161/230 (70.0)	46/230 (20.0)

Values are presented as n/N (%) where n is the number of samples in that category and N is the total number of informative samples. For the comparisons SMB–TE, SBM–ICM and TE–ICM, the reference samples were TE, ICM, and ICM, respectively.

## Data Availability

The data presented in this study is available on request from the corresponding author due to privacy restrictions to, in line with its codified nature, further protect the privacy of the participants’ clinical data.

## Supplementary Materials

The following supporting information can be downloaded at https://www.mdpi.com/article/10.3390/genes17040416/s1: Table S1. Detailed information on the ethical approvals for the 10 participating IVF centers. Table S2. Culture conditions (culture media and incubator used) by each of the 10 participating IVF centers. Table S3. Patient demographics and clinical background. Table S4. Ovarian stimulation and cycle characteristics. Table S5. Number of SBM samples analyzed per cycle and incidence of aneuploidies in SBM samples by age group. Table S6. Clinical outcomes obtained for the SETs performed. The information from all 441 SETs performed in the study is shown in Table S6A. The clinical information after excluding the transfers performed for patients with endometrial factor is shown in Table S6B (for all the transfers), S6C (cycles with patients’ own oocytes), S6D (cycles with donated oocytes). The data is presented in three groups, depending on the result obtained for the SBM. In all cases, only euploid blastocysts, determined by the TE biopsy, were transferred. Figure S1. Standards for Reporting of Diagnostic Accuracy Studies diagram for accuracy comparison between SBM and TE biopsies. The index test represented the SBM analyzed, and the reference standard represented the TE biopsy. The presence of aneuploidies in the TE biopsy is considered “condition present”, whereas a euploid TE result is considered “condition absent”. AF, amplification failure or no DNA detected; NI, non-informative. Figure S2. SBM informativity rate and SMB–TE ploidy concordance rate analyzed depending on female age (cycles with patients’ own oocytes). “n” refers to the number of samples included in each group. Figure S3. SBM informativity rate and SMB–TE ploidy concordance rate analyzed depending on embryo expansion degree (a) and ICM/TE quality (b) (cycles with patients’ own oocytes). *p*-values shown when statistically significant differences were observed. “n” refers to the number of samples included in each group. Figure S4. SBM informativity rate and SMB–TE ploidy concordance rate analyzed depending on culture conditions: incubator model (a) and medium brand (b) (cycles with patients’ own oocytes). *p*-values shown when statistically significant differences were observed. “n” refers to the number of samples included on each group. Figure S5. Incidence of aneuploidies per chromosome in TE biopsies and SBM samples.

## Abbreviations

The following abbreviations are used in this manuscript:

cfDNA	Cell-free DNA
ICM	Inner cell mass
niPGT-A	Non-invasive preimplantation genetic testing for aneuploidies
SBM	Spent blastocyst medium
SET	Single embryo transfer
TE	Trophectoderm
