# Supplementary material for: Can Cell-Free DNA in the Culture Medium Predict the Chromosomal Constitution of Preimplantation Embryos? Final Results from a Multicenter Study with 2539 Blastocysts
Source: Genes (Basel). 2026 Mar 31;17(4):416. doi: 10.3390/genes17040416 (PMC13116962; doi:10.3390/genes17040416)
Supplement: Supplementary file 1 [file genes-17-00416-s001.zip › genes-4153574-supplementary.pdf]

genes

Supplemental Material

Can Cell-Free DNA in the Culture Medium Predict the Chromosomal Constitution of Preimplantation Embryos? Final Results from a Multicenter Study with 2539 Blastocysts

Luis Navarro-Sánchez [1,*], Denny Sakkas [2], Nilo Frantz [3], Emilio de la Fuente Lucena [1], William Venier [4], Daria Maria Soscia [5,6], Gerardo Barroso [7], Claudio Bisioli [8], Michael DiMattina [9], Bilgen Teke [10], Luis Ernesto Escudero [11] and Carmen Rubio [1]

1 Research and Development Department, Igenomix, Part of Vitrolife Group, 46980 Paterna, Spain
2 IVIRMA Global Research Alliance, Waltham, MA 02451, USA
3 Nilo Frantz Reproductive Medicine, Porto Alegre 91330-000, Brazil
4 San Diego Fertility Center, San Diego, CA 92108, USA
5 IVIRMA Global Research Alliance, Genera, 00197 Roma, Italy
6 Department of Biomedicine and Prevention, University of Tor Vergata, 00133 Roma, Italy
7 Nascere, Mexico City 05120, Mexico
8 Pregna Medicina Reproductiva, Buenos Aires 1425, Argentina
9 Dominion Fertility, Arlington, VA 22203, USA
10 Bahçeci Health Group, 34394 Istanbul, Turkey
11 Inmater Fertilidad, Lima 15036, Peru

* Correspondence: luis.navarro@vitrolifegroup.com

Supplemental Material

Supplemental Table S1. Detailed information on the ethical approvals for the 10 participating IVF centers.

Ethics Committee	Date of approval	Code	Clinic (Country)
WCG IRB (Western Copernicus Group Institutional Review Board; formerly Western Institutional Review Board, or WIRB)	16/08/2018	20181919	Boston IVF (USA)[†]
WCG IRB (Western Copernicus Group Institutional Review Board; formerly Western Institutional Review Board, or WIRB)	24/08/2018	20181919	San Diego Fertility Center (USA)
WCG IRB (Western Copernicus Group Institutional Review Board; formerly Western Institutional Review Board, or WIRB)	04/08/2019	20181919	Dominion Fertility (USA)
Institutional review board from Clinica Valle Giulia	08(03/2018	Not provided	Genera (Italy)
Istanbul Bilim Üniversitesi Klinik Arastirmalari Ethics Committee	25/09/2018	B.08.6.YÖK.2.ÜS.0.05.0.06/2018/857	Bahçeci Health Group (Türkiye)
Institutional review board from the Centro de Educación Médica e Investigaciones Clínicas "Norberto Quirno"	07/11/2018	1171	Pregna Medicina Reproductiva (Argentina)
La Princesa Ethics Committee	26/07/2018	3514	IVF Spain (formerly ProcreaTec) (Spain)
Universidade Federal do Rio Grande do Sul e CONEP Ethics Committee	17/04/2019	03745318.1.0000.5347	Nilo Frantz e Centro de Reproduçao Humana (Brazil)[†]
Hospital ABC Ethics Committee	08/10/2018	IGX1-NIP-CS-18-02	Nascere (Mexico)
Comité Institucional de Ética en Investigación de la Universidad Peruana Cayetano Heredia	16/12/2019	19020	Inmater (Peru)

[†] The approved study protocol in these two centers also included the analysis of ICM biopsies on donated blastocysts. Accordingly, the informed consent forms provided to patients at both centers contained detailed information regarding the option to participate, ensuring that individuals could make an informed decision if they wish to do so.

Supplemental Table S2. Culture conditions (culture media and incubator used) by each of the 10 participating IVF centers.

Culture media	Incubator	Clinic (Country)
Single step - Irvine Scientific	MINC, Cook Medical	Boston IVF (USA)
Single step - Life Global	C-top, Labotec	San Diego Fertility Center (USA)
Single step - Life Global	MINC, Cook Medical	Dominion Fertility (USA)
Single step - Irvine Scientific	MINC, Cook Medical or K-System, CooperSurgical	Genera (Italy)
Single step - Irvine Scientific	MIRI, ESCO	Bahçeci Health Group (Türkiye)
Single step - Life Global or sequential - Sage	MIRI, ESCO or K-System, CooperSurgical	Pregna Medicina Reproductiva (Argentina)
Single step - Irvine Scientific	MINC, Cook Medical	IVF Spain (formerly ProcreaTec) (Spain)
Single step - Irvine Scientific	K-System, CooperSurgical	Nilo Frantz e Centro de Repro-duçao Humana (Brazil)
Single step - Vitrolife	ASTEC, Astec	Nascere (Mexico)
Single step - Life Global	K-System, CooperSurgical	Inmater (Peru)

Supplemental Table S3. Patient demographics and clinical background.

**Patient characteristics**	
No. of cycles (patients)	850 (716)
Mean female age (SD), y	36.8 (4.9)
Mean male age (SD), y	39.9 (6.0)
Mean no. of previous implantation failures (SD)	0.6 (1.3)
Mean no. of previous miscarriages (SD)	0.7 (1.2)
Mean no. of previous live births (SD)	0.3 (0.7)
Mean female BMI (SD), kg/m2	24.1 (4.3)
Previous PCO history (%)	80/739 (10.8)
Previous endometriosis history (%)	84/737 (11.4)
Primary PGT-A indication (%):	
AMA	553/850 (65.1)
RPL	35/850 (4.1)
RIF	33/850 (3.9)
GP	66/850 (7.8)
GS	9/850 (1.1)
PTP	22/850 (2.6)
SMF	30/850 (3.5)
Mixed causes	26/850 (3.1)
OD	76/850 (8.9)

Values are presented as n/N (%) unless indicated otherwise. Percentages were calculated considering the number of cycles with informative data for each variable. AMA, advanced maternal age; BMI, body mass index; GP, good prognosis; GS, gender selection; OD, ovum donation; PCO, polycystic ovary; PGT-A, preimplantation genetic testing for aneuploidy; PTP, previous trisomy pregnancy; RIF, repetitive implantation failure; RPL, recurrent pregnancy loss; SD, standard deviation; SMF, severe male factor.

Supplemental Table S4. Ovarian stimulation and cycle characteristics.

**Cycle characteristics**	
ICSI cycles (%)	763/850 (89.8)
IVF cycles (%)	67/850 (7.9)
ICSI-IVF cycles (%)	20/850 (2.4)
Type of gonadal suppression (%):	
Antagonist protocol	804/823 (97.7)
Long-agonist protocol	9/823 (1.1)
Short-agonist protocol	10/823 (1.2)
Type of stimulation protocol (%):	
Clomiphene citrate	2/838 (0.2)
FSH only	138/838 (16.5)
FSH + hMG	297/838 (35.4)
FSH + LH	378/838 (45.1)
hMG	23/838 (2.7)
Type of ovulation triggering (%)	
Double triggering	92/838 (11.0)
GnRH agonist	448/838 (53.5)
hCG	298/838 (35.6)
Sperm origin (%):	
Donated	41/849 (4.8)
Ejaculated	783/849 (92.2)
Epidydimal	13/849 (1.5)
Testicular	12/849 (1.4)
Mean stimulation days (SD)	10.7 (1.7)
Mean number of MII oocytes (SD)	10.1 (7.0)
Mean number of 2PN oocytes (SD)	7.9 (5.9)

Values are presented as n/N (%) unless indicated otherwise. Percentages were calculated considering the number of cycles with informative data for each variable. 2PN, 2 pronuclei; FSH, follicle-stimulating hormone; GnRH, gonadotropin-releasing hormone; hCG, human chorionic gonadotropin hormone; hMG, human menopausal gonadotropin; ICSI, intracytoplasmic sperm injection; IVF, in vitro fertilization; LH, luteinizing hormone; MII, metaphase II; SD, standard deviation.

Supplemental Table S5. Number of SBM samples analyzed per cycle and incidence of aneuploidies in SBM samples by age group.

Female Age (y)	≤30	31-35	36-38	39-40	41-44
Mean number of SBM samples per cycle	4.6	3.6	2.7	2.3	1.9
Incidence of aneuploidies in SBM samples (%)	34.7	39.2	53.1	61.5	74.5

The incidence of aneuploidies was calculated by dividing the number of aneuploid samples by the total number of informative samples in each age group.

Supplemental Table S6. Clinical outcomes obtained for the SET performed. The information from all 441 SET performed in the study is shown in Table S6A. The clinical information after excluding the transfers performed for patients with endometrial factor is shown in Table S6B (for all the transfers), 6C (cycles with patient's own oocytes), 6D (cycles with donated oocytes). The data is presented in three groups, depending on the result obtained for the SBM. In all cases, only euploid blastocysts, determined by the TE biopsy, were transferred.

A)

	**Euploid TE / Euploid SBM**	**Euploid TE / Aneuploid SBM**	**Euploid TE / Non-informative SBM**
No. of transfers	288	95	58
Mean female age (SD), y	35.3 (4.9)	34.4 (5.2)	35.8 (4.7)
Positive pregnancy rate	172/288 (59.7)	61/95 (64.2)	30/58 (51.7)
Clinical pregnancy rate	150/288 (52.1)	55/95 (57.9)	27/58 (46.6)
Miscarriage rate	22/150 (14.7)	14/55 (25.5)	3/27 (11.1)
Live birth rate	128/288 (44.4)	41/95 (43.2)	24/58 (41.4)

B)

	**Euploid TE / Euploid SBM**	**Euploid TE / Aneuploid SBM**	**Euploid TE / Non-informative SBM**
No. of transfers	231	73	52
Mean female age (SD), y	35.4 (5.0)	34.5 (5.2)	35.7 (4.8)
Positive pregnancy rate	145/231 (62.8)	47/73 (64.4)	28/52 (53.8)
Clinical pregnancy rate	133/231 (57.6)	43/73 (58.9)	25/52 (48.1)
Miscarriage rate	19/133 (14.3)	11/43 (25.6)	3/25 (12.0)
Live birth rate	114/231 (49.4)	32/73 (43.8)	22/52 (42.3)

C)

	**Euploid TE / Euploid SBM**	**Euploid TE / Aneuploid SBM**	**Euploid TE / Non-informative SBM**
No. of transfers	203	62	44
Mean female age (SD), y	36.8 (3.5)	36.1 (3.5)	37.2 (3.1)
Positive pregnancy rate	124/203 (61.1)	38/62 (61.3)	25/44 (56.8)
Clinical pregnancy rate	115/203 (56.7)	36/62 (58.1)	22/44 (50.0)
Miscarriage rate	15/115 (13.0)	8/36 (22.2)	3/22 (13.6)
Live birth rate	100/203 (49.3)	28/62 (45.2)	19/44 (43.2)

D)

	**Euploid TE / Euploid SBM**	**Euploid TE / Aneuploid SBM**	**Euploid TE / Non-informative SBM**
No. of transfers	28	11	8
Mean female age (SD), y	25.6 (2.6)	25.4 (3.2)	27.3 (3.7)
Positive pregnancy rate	21/28 (75.0)	9/11 (81.8)	3/8 (37.5)
Clinical pregnancy rate	18/28 (64.3)	7/11 (63.6)	3/8 (37.5)
Miscarriage rate	4/18 (22.2)	3/7 (42.9)	0
Live birth rate	14/28 (50.0)	4/11 (36.4)	3/8 (37.5)

Values for positive pregnancy rate, clinical pregnancy rate, miscarriage rate and live birth rate are presented as n/N (%). SD, standard deviation. Y, years.

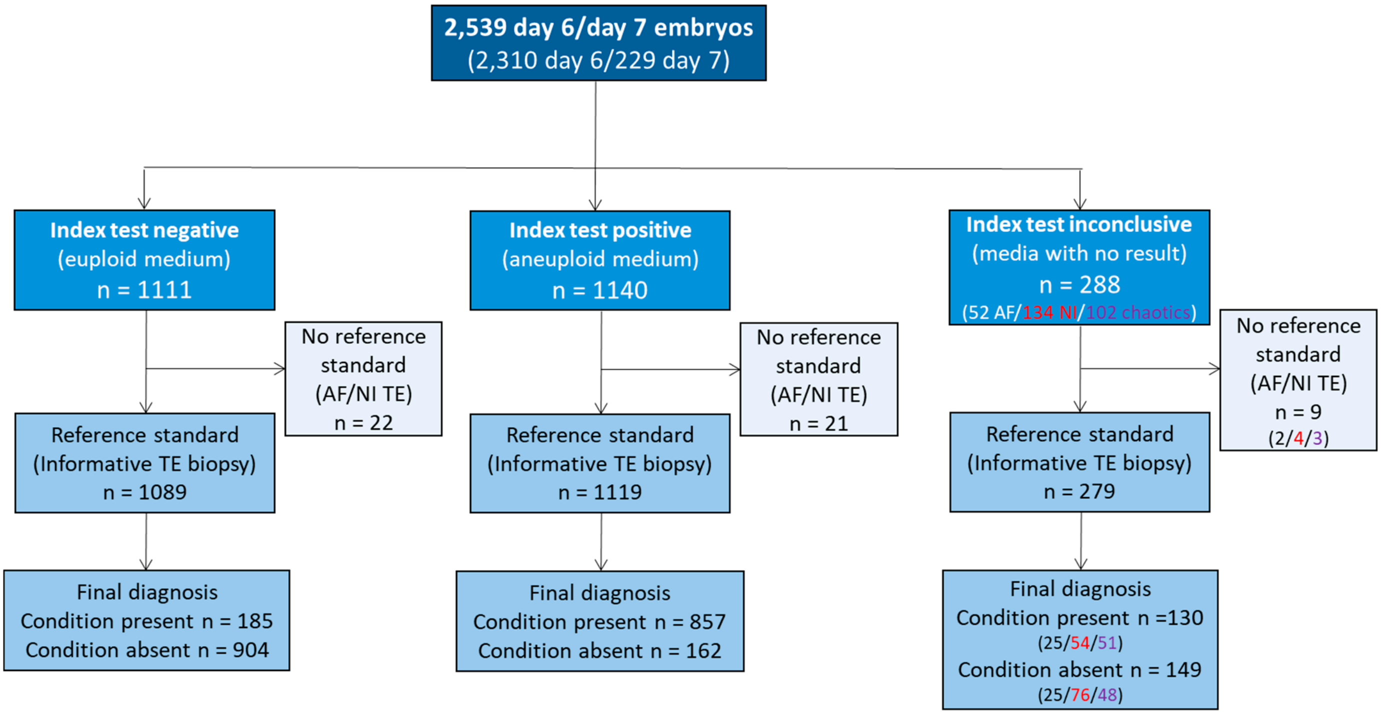

Supplemental Figure S1. Standards for Reporting of Diagnostic Accuracy Studies diagram for accuracy comparison between SBM and TE biopsies. The index test represented the SBM analyzed, and the reference standard represented the TE biopsy. The presence of aneuploidies in the TE biopsy is considered "condition present", whereas a euploid TE result is considered "condition absent". AF, amplification failure or no DNA detected; NI, non-informative.

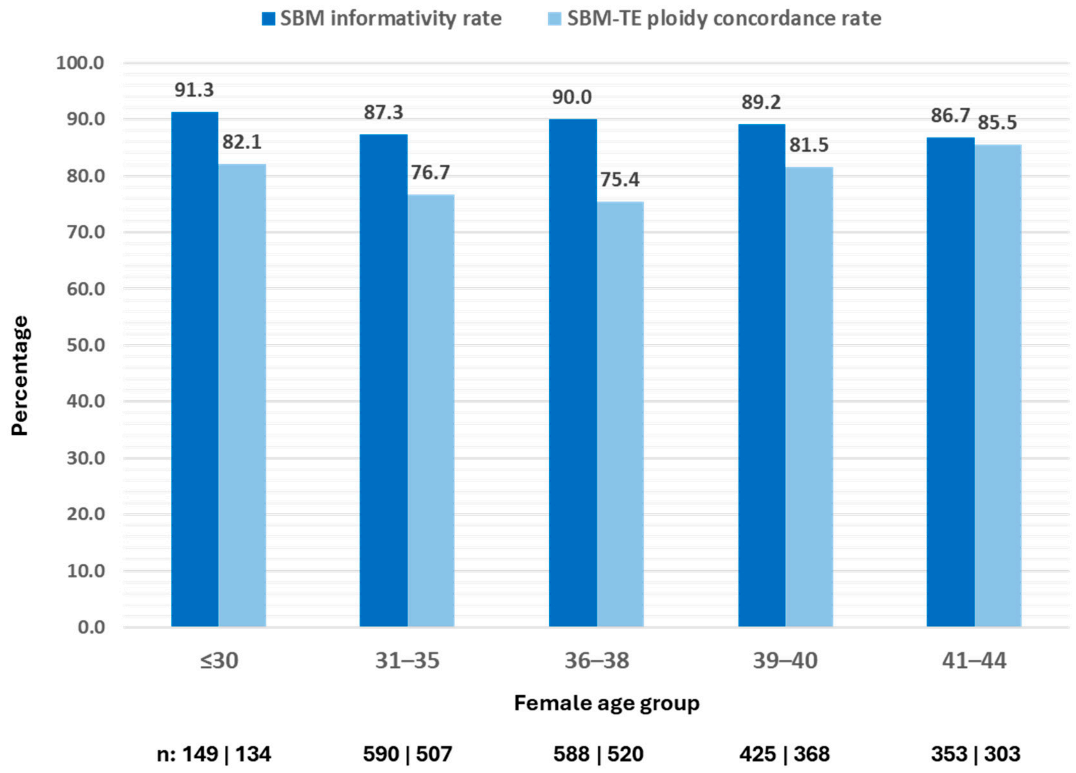

Supplemental Figure S2. SBM informativity rate and SBM-TE ploidy concordance rate analyzed depending on female age (cycles with patient's own oocytes). "n" refers to the number of samples included in each group.

(**a**)

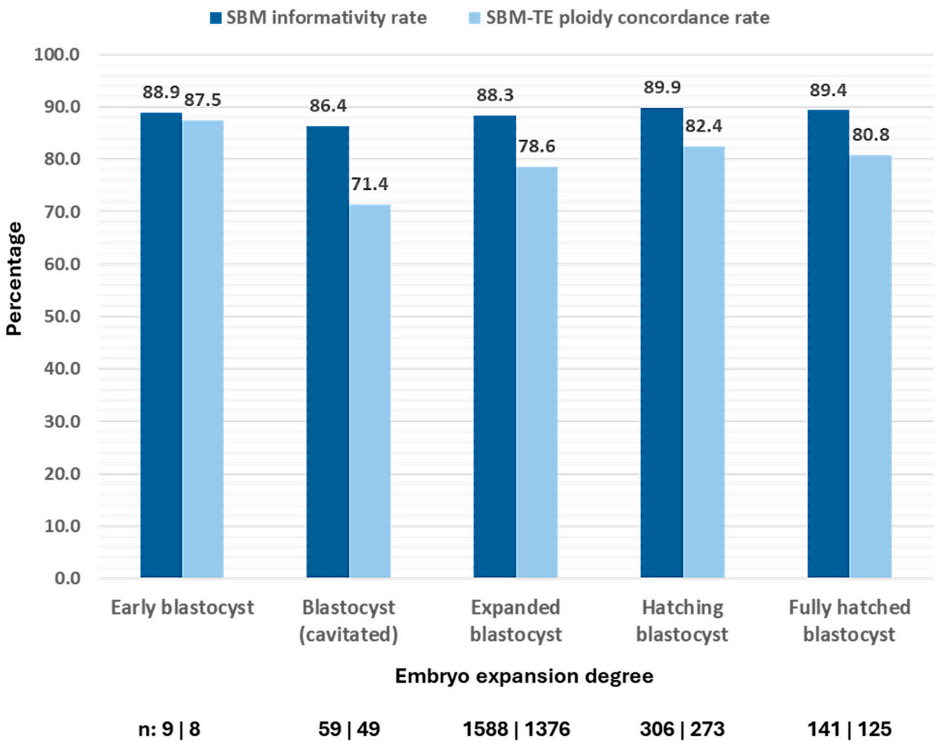

(**b**)

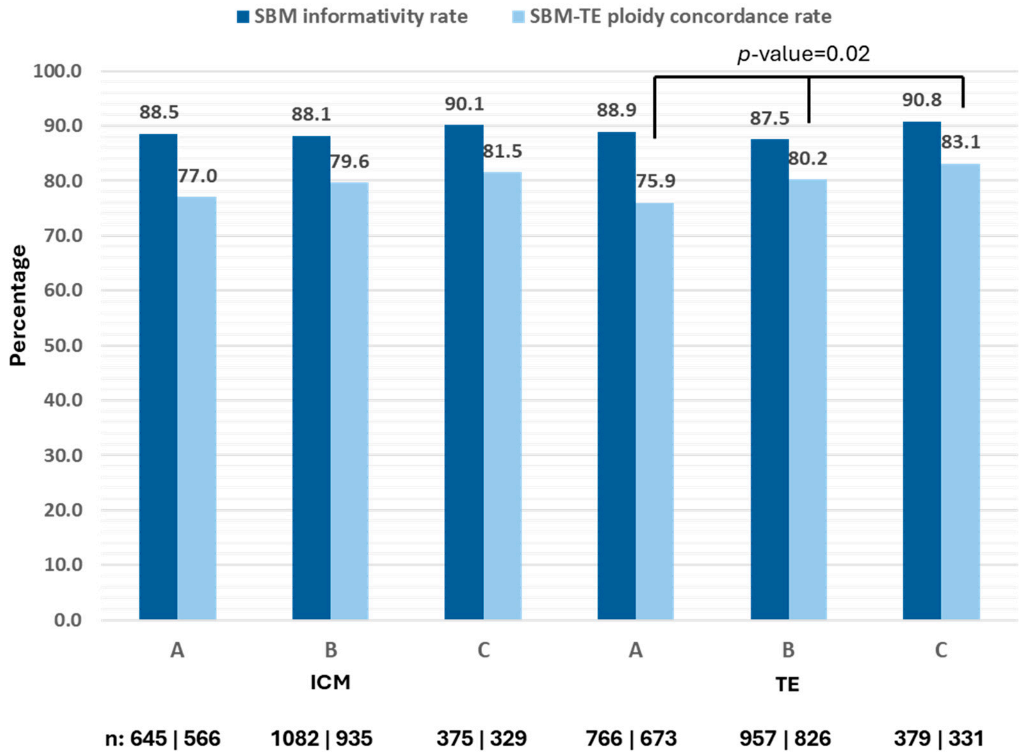

Supplemental Figure S3. SBM informativity rate and SBM-TE ploidy concordance rate analyzed depending on embryo expansion degree (a) and ICM/TE quality (b) (cycles with patient's own oocytes). *p*-values shown when statistically significant differences were observed. "n" refers to the number of samples included in each group.

(**a**)

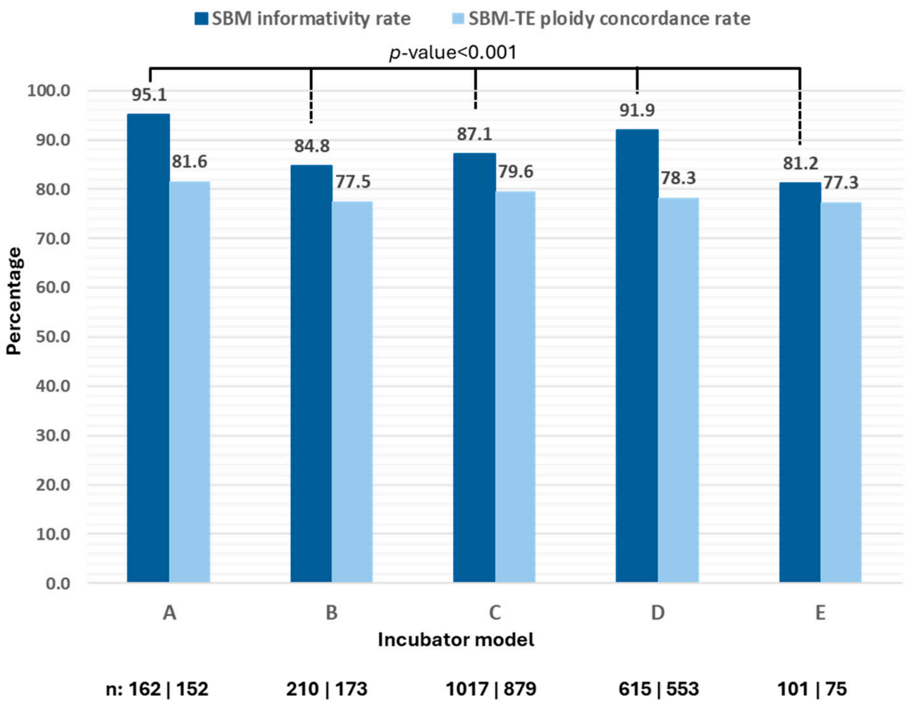

(**b**)

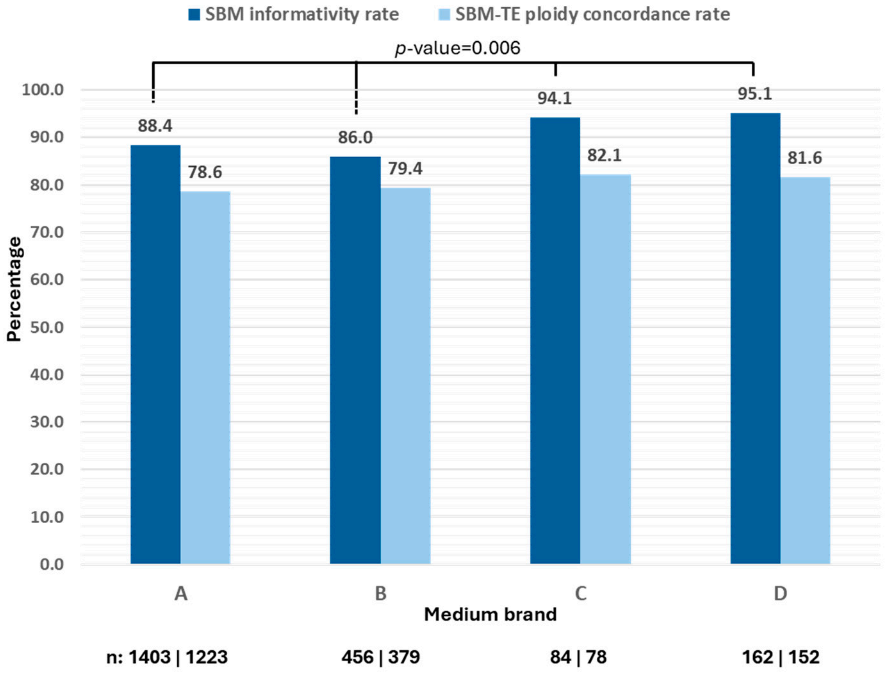

Supplemental Figure S4. SBM informativity rate and SBM-TE ploidy concordance rate analyzed depending on culture conditions: incubator model (a) and medium brand (b) (cycles with patient's own oocytes). *p*-values shown when statistically significant differences were observed. "n" refers to the number of samples included on each group.

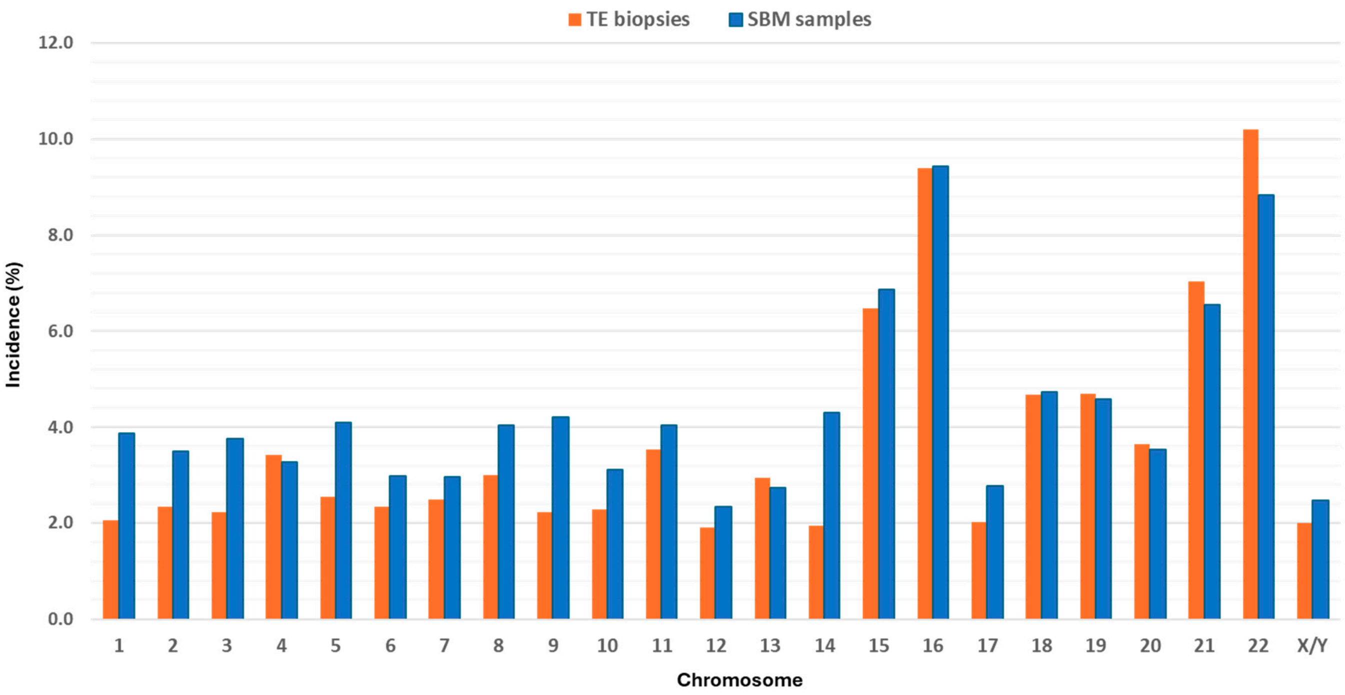

Supplemental Figure S5. Incidence of aneuploidies per chromosome in TE biopsies and SBM samples.

Disclaimer/Publisher's Note: The statements, opinions and data contained in all publications are solely those of the individual author(s) and contributor(s) and not of MDPI and/or the editor(s). MDPI and/or the editor(s) disclaim responsibility for any injury to people or property resulting from any ideas, methods, instructions or products referred to in the content.
